# The anoikis-related gene signature predicts survival and correlates with immune infiltration in osteosarcoma

**DOI:** 10.18632/aging.205411

**Published:** 2024-01-12

**Authors:** Junqing Li, Hui Wang, Feiran Wu, Jie Yao, Huimin Zhu, Meng Zhang

**Affiliations:** 1 Minimally Invasive Spinal Surgery Center, Luoyang Orthopedic-Traumatological Hospital of Henan Province (Henan Provincial Orthopedic Hospital), Zhengzhou 450018, China; 2 Department of Orthopedics, Henan Provincial People’s Hospital, Zhengzhou University People’s Hospital, Henan University People’s Hospital, Zhengzhou 450003, China

**Keywords:** osteosarcoma, anoikis, tumor immunity, OGT

## Abstract

Anoikis is essential for the progression of many malignant tumors. However, the understanding of anoikis’ roles in osteosarcoma remains scarce. This study conducted an extensive bioinformatics analysis to identify anoikis-related genes (ARGs), developed ARGs modeles for predicting OS and RFS, and evaluated the effect of these ARGs on osteosarcoma cell migration and invasion. The GSE16088 and GSE28425 datasets provided the differentially expressed genes (DEGs). The prognostic significance and functions of these DEGs were systematically investigated using several bioinformatics techniques. Transwell assays were conducted to determine the effect of OGT on osteosarcoma cell migration and invasion. Seven genes were identified as hub genes, including *FN1, CD44, HRAS, TP53, PPARG, CTNNB1*, and *VEGFA*, while 71 ARGs were identified as DEGs. Four ARGs-*BRMS, COL4A2, FGF2*, and *OGT*-were used to develop an RFS-predicting model, whereas seven ARGs-*CD24, FASN, MMP2, EIF2AK3, ID2, PPARG*, and *PIK3R3*-were used to develop an OS-predicting model in patients with osteosarcoma. In both the training and validation cohorts, high-risk group patients had significantly shorter OS and RFS duration than low-risk group patients. Furthermore, using the aforementioned ARGs, we developed clinically applicable nomograms for OS and RFS prediction. The proportion of tumor-infiltrating immune cells was significantly linked to risk scores. *In vitro* experiments revealed that knocking down OGT significantly inhibited the ability of MG63 and U2OS cells to invade and migrate. ARG-based gene signatures reliably predicted RFS and OS in osteosarcoma, and OGT showed promise as a potential biomarker. These findings contribute to a better understanding of ARGs’ prognostic roles in osteosarcoma.

## INTRODUCTION

Osteosarcoma is a prevalent malignant bone tumor that affects adolescents [1]. Osteosarcoma treatment includes complete surgical resection and multi-agent chemotherapy [1]. Although these treatments have improved survival in patients with osteosarcoma, the prognosis of unresectable or recurrent osteosarcomas is still unsatisfactory [2]. Therefore, it is urgently needed to seek novel biomarkers and elucidate the possible mechanisms involved in developing new therapeutic strategies and improving survival.

Anoikis, a mode of programmed cell death induced by insufficient cell-matrix interactions, may prevent the invasion and metastasis of cancer cells [3]. Anoikis is attracting more and more attention from researchers since resistance to anoikis is similar to two features of tumor metastatic—anchorage-independent growth and epithelial-mesenchymal transition [4]. Anoikis is crucial in tumor progression. In Ewing sarcoma, the inactivation of *IL1RAP* triggered anoikis and inhibited the dissemination of tumor cells [5]. In gastric cancer, nuclear *MYH9*-induced *CTNNB1* upregulation promoted cancer cell anoikis resistance and metastasis [6]. In hepatocellular carcinoma, *IQGAP1* promoted anoikis resistance and metastasis through activation of Src/FAK signaling [7]. In lung cancer, *SPIB* promoted anoikis resistance via elevated autolysosomal process [8]. *BMP4* enhanced anoikis resistance and chemoresistance of cancer cells in breast cancer through canonical BMP signaling [9]. These findings suggest that anoikis and anoikis-related genes (ARGs) are crucial for tumor progression and metastasis. ARGs also play important roles in osteosarcoma metastasis [10, 11], but we know only the tip of the iceberg. Therefore, a detailed investigation into the roles of ARGs and anoikis will aid in the development of new therapeutic approaches and prolong the survival duration of osteosarcoma patients.

This study used the TCGA, TARGET, and GEO databases for obtaining clinical and mRNA expression data on osteosarcoma. Hub genes and differentially expressed genes (DEGs) were identified. This study then developed and validated ARG-based models for predicting relapse-free survival (RFS) and overall survival (OS). The link between the risk score and tumor immunity was investigated. Finally, this study’s findings were validated using *in vitro* experiments.

## MATERIALS AND METHODS

### Data processing

The gene symbols for 434 ARGs were obtained from the study by Chen S et al. [12]. The gene expression profiles from GSE16088 [13] and GSE28425 [14] datasets were used to identify DEGs using the “limma” R package [15]. The “sva” and “limma” R packages were used to integrate and standardize the GSE16088 and GSE28425 datasets. DEGs were defined as those with p <0.05 and log2|fold change| values >1. The “ggplot2” [16] and “pheatmap” [17] packages were used to generate volcano plots and heatmaps, respectively.

Data for the OS model were obtained from the TCGA and TARGET databases for training cohorts, and the validation cohorts’ clinical data and gene expression profiles were obtained from the GSE16091 [13] and GSE21257 [18] datasets. Integration and standardization of GSE16091 and GSE21257 datasets were implemented using the “sva” and “limma” R packages. For the RFS model, the gene expression profiles and clinical information for the training cohort were extracted from the GSE39058 [19] dataset, while the validation cohorts were downloaded from the TARGET and TCGA databases.

### Identification of hub genes

The previously described method [20] was used to identify hub genes. The STRING database [21] was used to retrieve data on DEG protein-protein interactions (PPI). Using Cytoscape 3.7.2 software, the PPI network was developed and visualized. Candidate hub genes were identified using the three methods, MCODE, degree, and betweenness, and the hub genes were identified based on the overlap of the three methods’ results.

### Prognostic model construction and validation

First, the OS prognostic model was constructed and validated. TCGA and TARGET data on mRNA expression and clinical information were collated and combined. The “survival” package was then used to perform Kaplan-Meier (KM) analysis and generate survival curves. Candidate model genes were those with p <0.05. The “glmnet” R package was then used to perform the least absolute shrinkage and selection operator (LASSO) Cox regression analysis to construct the OS model. Each patient’s risk score was also calculated. The risk score formula was as follows: RS= ∑Coef_genei_ x Exp_genei_. Patients were classified into high-risk and low-risk groups based on their median risk scores. The survival curves of the two groups were plotted to compare OS. The “survivalROC” R package was used to plot the receiver operating characteristic (ROC) curve to evaluate the OS model’s predictive capability. Similarly, risk scores were calculated for patients in the validation (GSE16091 and GSE21257) cohort, and survival curves and ROC curves were visualized.

We used the same methods as above to construct the RFS model. The training cohort was GSE39058, and the validation cohort was TCGA. Finally, we developed nomograms using the “rms” package to predict OS and RFS more intuitively and conveniently.

### GO enrichment, KEGG pathway, GSVA analysis and biological networks

We used the ‘clusterProfiler” R package to conduct GO and KEGG pathway analyses to further investigate the potential functions and mechanisms of hub genes and prognosis-related genes [22]. Then, we performed GSVA analyses to explore the reasons for the different prognosis of patients with high or low risk as previously described [23]. In addition, Transcription factor (TF)-microRNA (miRNA) coregulatory networks of prognosis-related and hub genes were established using NetworkAnalyst 3.0.

### Immune scores and tumor-infiltrating immune cell analysis

The ESTIMATE algorithm calculated immune and stromal scores for the training (TCGA-TAEGET) cohort and validation (GSE16091 and GSE21257) cohort samples. Tumor-infiltrating immune cells (TIICs) were assessed in the training (TCGA-TAEGET) cohort and validation (GSE16091 and GSE21257) cohort using the CIBERSORT online tool [24].

### Cell culture and transfection

From the Cell Bank of the Chinese Academy of Sciences (Shanghai, China), MG63 and U2OS cell lines were purchased. DMEM with 10% FBS (Biological Industries, Shanghai, China) was used to culture both cell lines. GeneChem (Shanghai, China) provided OGT-targeting siRNA and negative control siRNA (si-nc). Supplementary Table 1 provides comprehensive details about the si-1 and si-2 sequences. Lipofectamine 3000 (Invitrogen, Carlsbad, CA, USA) was used to transfect the cells with the siRNAs.

### Quantitative real-time PCR (RT-PCR) and Western blotting (WB)

Quantitative RT-PCR and WB were carried out as previously described [20]. RT-PCR reagents were purchased from Accurate Biology (Changsha, China), which included the SYBR Green Premix Pro Taq HS qPCR Kit, AG RNAex Pro Reagent, and Evo M-MLV RT Premix Kit. Supplementary Table 1 shows the primer sequences. Beyotime Biotechnology (Shanghai, China) provided WB reagents, such as BeyoECL Plus and RIPA buffer Kit. Proteintech (Wuhan, China) supplied anti-OGT (11576-2-AP) and anti-GAPDH (10494-1-AP) antibodies.

### Cell migration and invasion assays

Transwell assays were performed as previously described [25] to assess the effects of OGT expression on MG63 and U2OS cell migration and invasion. Finally, cells that migrated into the lower chamber were counted using a microscope in five different fields.

### Statistical analysis

Most bioinformatics and statistical analyses in this study were performed using R software (version 4.0.0), including integration and normalization of mRNA expression data, LASSO Cox regression analysis, survival analysis, ROC analysis, CIBERSORT, and ESTIMATE. The mean ± standard deviation of three independent experiments was used to represent all quantitative data obtained from *in vitro* experiments. GraphPad Prism 8.0 (GraphPad, La Jolla, CA, United States) was used to analyze differences across three groups using one-way ANOVA. p <0.05 denoted a statistical significance level.

### Data availability statement

All datasets presented in this study are included in the article Supplementary Material.

## RESULTS

### DEGs and hub genes in osteosarcoma

**Figure 1:**
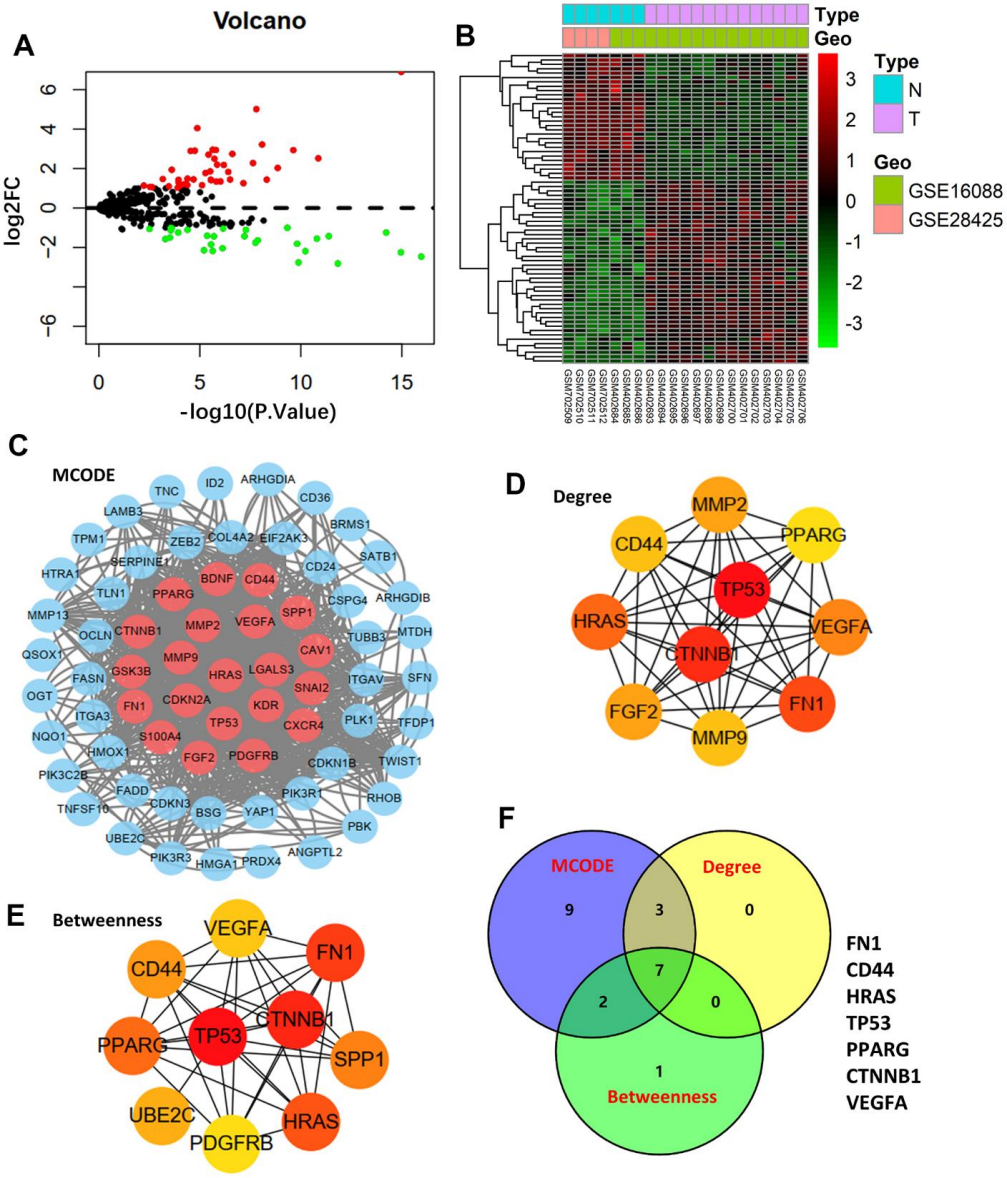
**Differentially expressed genes and hub genes.** (**A**) Volcano plot of 434 ARGs in GSE16088 and GSE28425 datasets. (**B**) Heatmap of DEGs in GSE16088 and GSE28425 datasets. (**C**) Protein-protein interaction (PPI) network of 71 DEGs and the critical module. Identifying the first 10 ARGs and constructing the corresponding PPI network using the degree (**D**) and betweenness (**E**) topological method. (**F**) Venn calculation is applied to identify seven hub ARGs.

The GSE16088 and GSE28425 datasets of normal bone and tumor tissues were used to identify differentially expressed ARGs. Figure 1A, 1B show the Volcano plot and heatmap of ARGs in GSE16088 and GSE28425, respectively. Finally, 71 ARGs out of 434 ARGs were identified as DEGs (Supplementary Table 2).

After mapping the 71-DEG PPI network with STRING, the Cytoscape MCODE plug-in was used to reconstruct the network (Figure 1C). The first 10 genes were chosen, and the degree and betweenness topological approaches were used to construct the associated PPI network (Figure 1D, 1E). Seven genes—*FN1*, *CD44*, *HRAS*, *TP53*, *PPARG*, *CTNNB1*, and *VEGFA*—were shown to be hub genes by crossing the three methods’ results (Figure 1F).

### Prognostic model construction and validation

**Figure 2:**
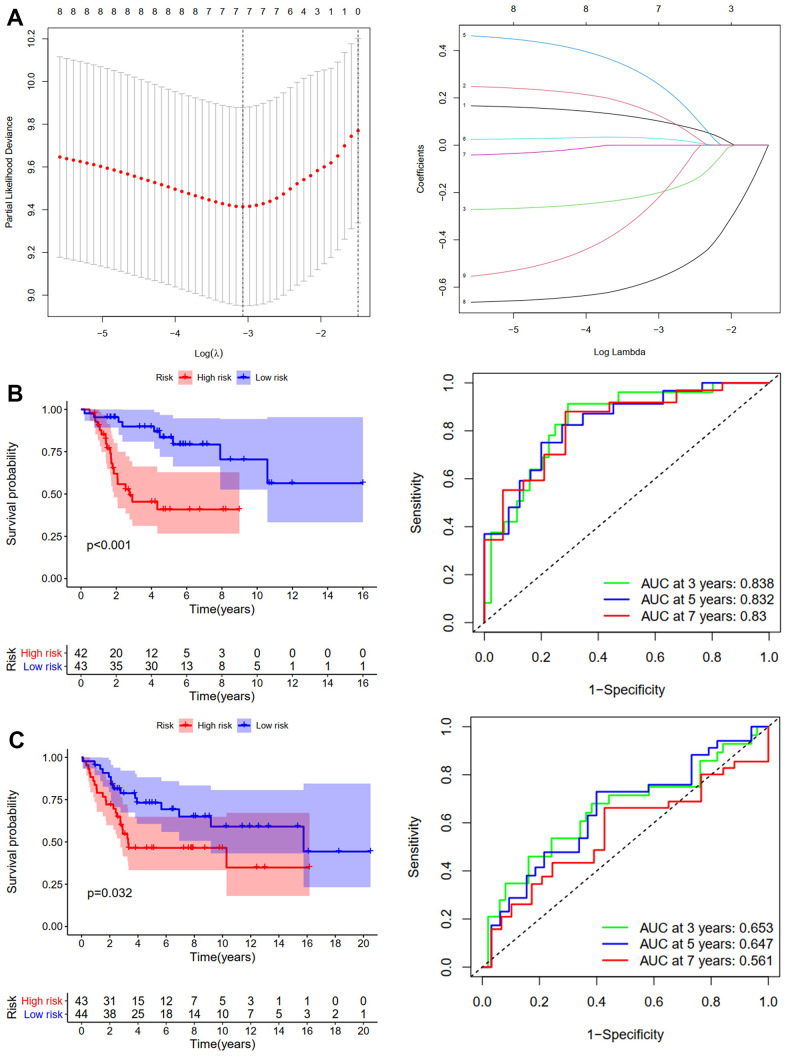
**Development and validation of the OS-prediction model for osteosarcoma.** (**A**) The least absolute shrinkage and selection operator (LASSO) method of ARGs is associated with prognosis. Survival curve and ROC curve for low- and high-risk subgroups in the training cohort (TCGA-TAGET) (**B**) and the validation cohort (GSE16091 and GSE21257) (**C**).

First, we conducted KM analysis to investigate how DEGs affected the training (TCGA-TARGET) cohort’s OS before analyzing ARGs’ effects on osteosarcoma patients’ prognoses. The results revealed that OS was substantially correlated with nine candidate ARGs (Supplementary Figure 1). Subsequently, the OS model was constructed using LASSO regression analysis. Figure 2A shows the corresponding confidence interval for each lambda and each gene’s lambda-valued coefficient trajectory. Seven signature genes were chosen for the OS model. The following formula was used to calculate the risk score: (0.106*Exp CD24) + (0.135*Exp FASN) + (-0.206*Exp MMP2) + (0.272*Exp EIF2AK3) + (0.029*Exp ID2) + (-0.569*Exp PPARG) + (-0.243*Exp PIK3R3). The training cohort’s median risk score classified patients into low- and high-risk groups. According to the KM analysis results, patients in the high-risk group had considerably worse (p <0.001, Figure 2B). We next performed ROC analyses to evaluate aforementioned ARGs’ predictive capabilities. The outcomes showed that the OS predictive model’s area under the ROC curve (AUC) values were 0.838, 0.832, and 0.83 over 3, 5, and 7 years, respectively (Figure 2B). The GSE16091 and GSE21257 datasets were then used for verification analysis. Patients at high risk had shorter OS duration, in line with the findings of the training cohort (p <0.05, Figure 2C). Over 3, 5, and 7 years, the OS model’s AUCs were 0.653, 0.647, and 0.561, respectively (Figure 2C).

**Figure 3:**
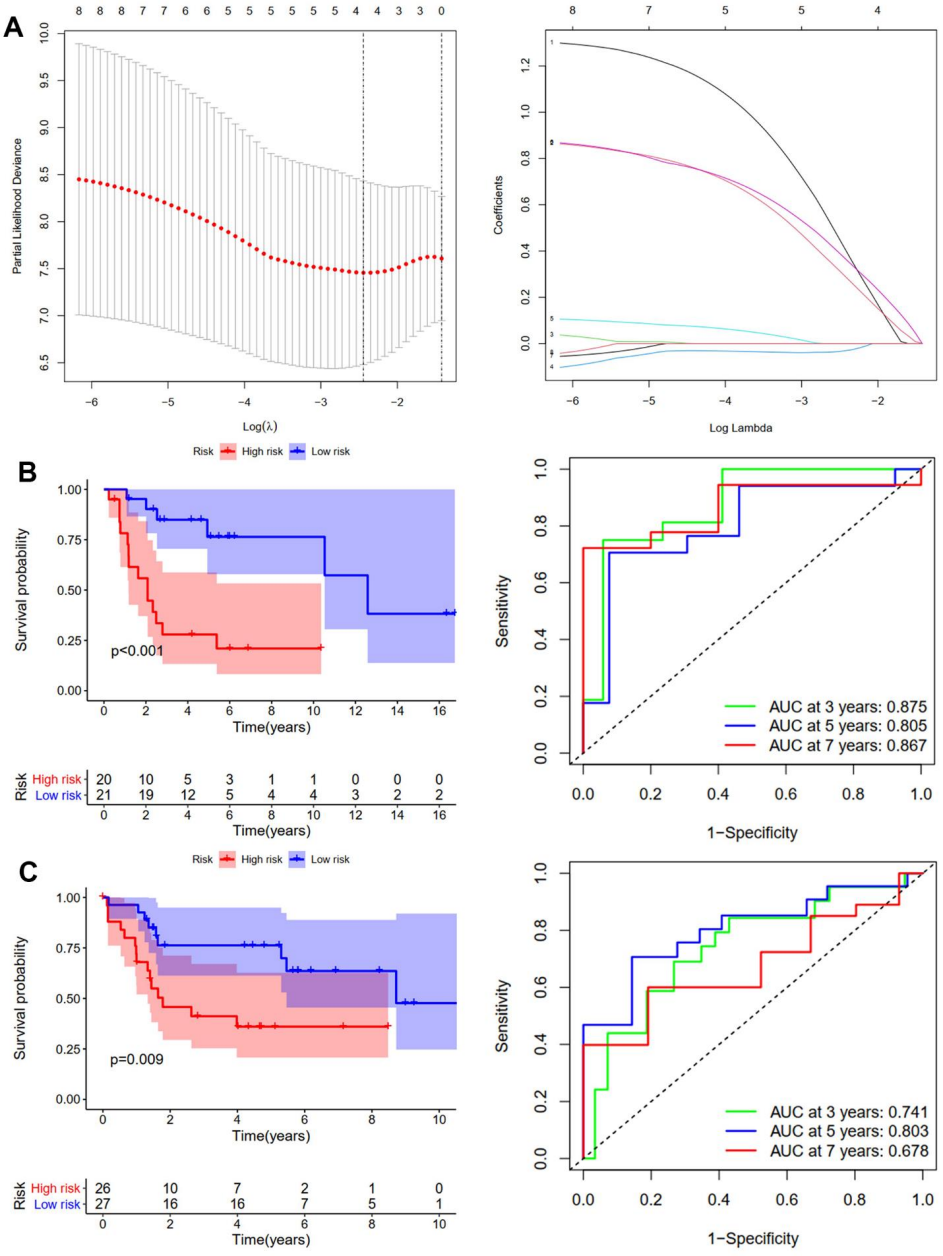
**Development and validation of the RFS-prediction model for osteosarcoma.** (**A**) The least absolute shrinkage and selection operator (LASSO) method of ARGs is associated with prognosis. Survival curve and ROC curve for low- and high-risk subgroups in the training cohort (GSE39058) (**B**) and the validation cohort (TCGA-TAGET) (**C**).

We used the same method to construct the RFS predictive model. GSE39058 sets were used as the training cohort. Eight candidates’ ARGs were strongly related to RFS, according to KM analysis (Supplementary Figure 2). The RFS model was constructed using LASSO regression analysis. Figure 3A shows the corresponding confidence interval for each lambda and each gene’s lambda-valued coefficient trajectory. Four signature genes were chosen for developing the RFS model. The following formula calculated the risk score: (0.416*Exp BRMS1) + (0.295*Exp COL4A2) + (-0.032*Exp FGF2) + (0.372*Exp OGT). The training cohort’s median risk score divided patients into low- and high-risk groups. According to the KM analysis results, patients in the high-risk group had considerably worse RFS (p <0.001, Figure 3B). Over 3, 5, and 7 years, the RFS model’s AUCs were 0.875, 0.805, and 0.867, respectively (Figure 3B). The validation cohort was then created using the TCGA-TARGET sets. Patients in the high-risk group had shorter RFS duration, which was consistent with the training cohort results (p =0.009, Figure 3C). Over 3, 5, and 7 years, the OS model’s AUCs were 0.741, 0.803, and 0.678, respectively (Figure 3C). The risk score and clinical parameters’ prognostic value were also assessed using univariate and multivariate Cox analyses. The findings revealed that patients in the TCGA-TARGET dataset had an independent risk score for OS (Supplementary Figure 3A) and RFS (Supplementary Figure 3B). In summary, osteosarcoma patients’ outcomes were accurately predicted by the OS and RFS models.

### Building predictive nomograms

**Figure 4:**
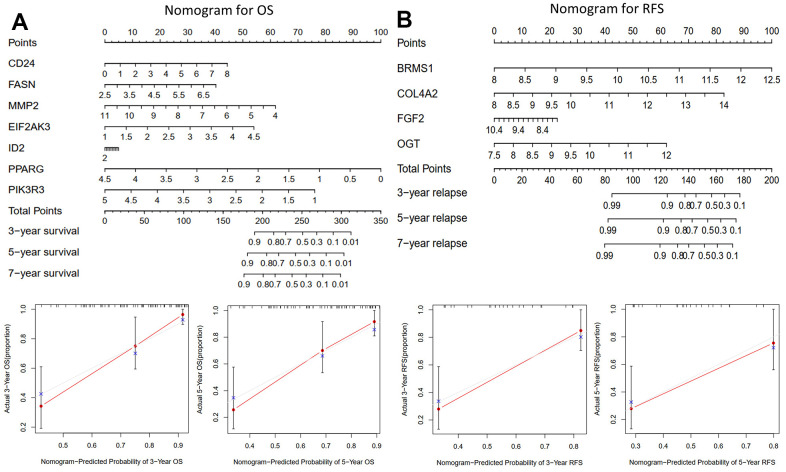
**The nomograms for predicting 3-, 5-, and 7-year OS and RFS of the training cohort.** (**A**) Nomogram for predicting 3- and 5-year OS in the training cohort (TCGA-TAGET) and calibration plots. (**B**) Nomogram for predicting 3- and 5-year RFS in the training cohort (GSE39058) and calibration plots.

Nomograms (Figure 4A, 4B) were constructed to enable us to better predict osteosarcoma patients’ OS and RFS using prognosis-related ARGs. Each gene was assigned a corresponding point on the point scale. According to each patient’s predicted 3-, 5-, and 7-year survival rates, the points were then added up to determine the total score for each patient.

### Potential functions and mechanisms

**Figure 5:**
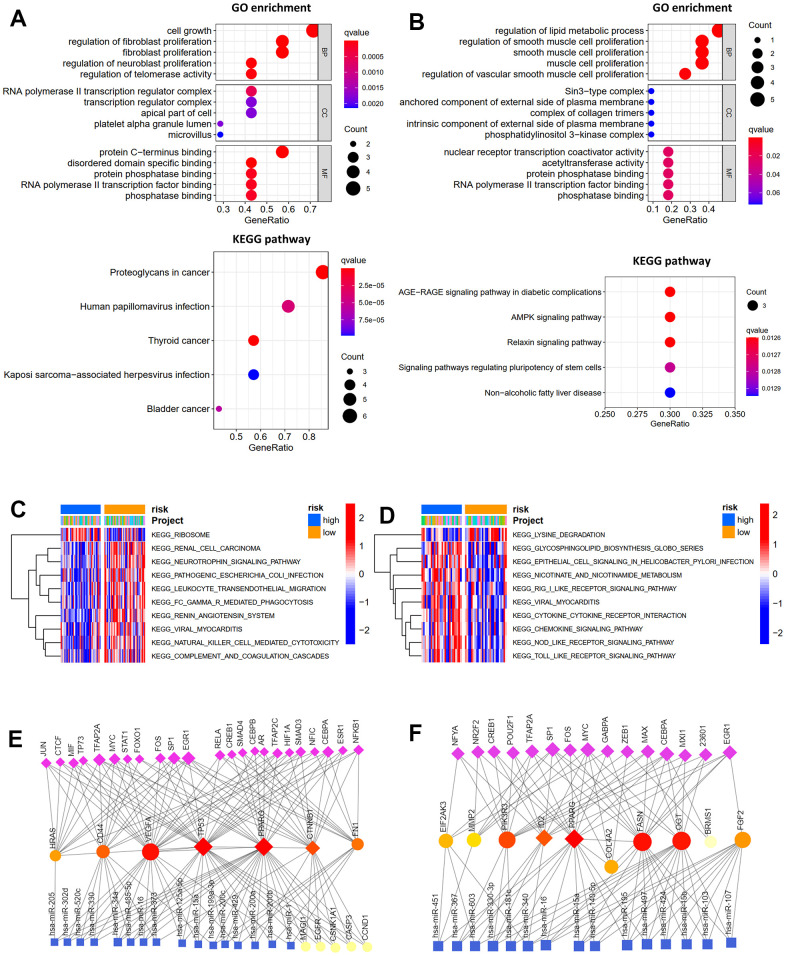
**Function enrichment analysis.** GO function enrichment and KEGG pathway analysis of hub genes (**A**) and prognosis-related ARGs (**B**). GSVA analysis for OS (**C**) and RFS (**D**) in low- and high-risk subgroups of TCGA cohort. Transcription factor-miRNA coregulatory network of hub genes (**E**) and prognosis-related ARGs (**F**).

GO and KEGG analyses of the hub and prognosis-related ARGs were carried out using the “clusterProfiler” R package to investigate their functions and possible mechanisms in osteosarcoma. As shown in Figure 5A, for GO enrichment analysis, the seven hub ARGs were enriched in cell growth, fibroblast proliferation, transcription regulator complex, protein-C terminus binding, and phosphatase binding. The prognosis-related ARGs were enriched in lipid metabolic process and smooth muscle cell proliferation (Figure 5B). For KEGG pathway analysis, the seven hub ARGs were enriched in virus infection and some cancers (Figure 5A). The prognosis-related ARGs were enriched in AGE-RAGE, AMPK, and Relaxin signaling pathways. (Figure 5B). Then, GSVA analyses were performed to explore the reasons for the different prognosis of patients with high or low risk. For OS model, the Ribosome pathway was activated primarily in high-risk patients, whereas low-risk patients mainly activated other pathways, such as leukocyte transendothelial migration, natural killer cell mediated cytotoxicity, and complement and coagulation cascades (Figure 5C). For RFS model, high-risk subgroups mainly activated cytokine- cytokine receptor interaction pathway, chemokine signaling pathway, NOD like receptor signaling pathway and TOLL like receptor signaling pathway, whereas low-risk subgroups mainly activated lysine degradation pathway (Figure 5D). Furthermore, the TF-miRNA coregulatory network of the seven hub ARGs (Figure 5E) and prognosis-related ARGs (Figure 5F) was constructed using NetworkAnalyst.

### Immune infiltration differences between risk groups

**Figure 6:**
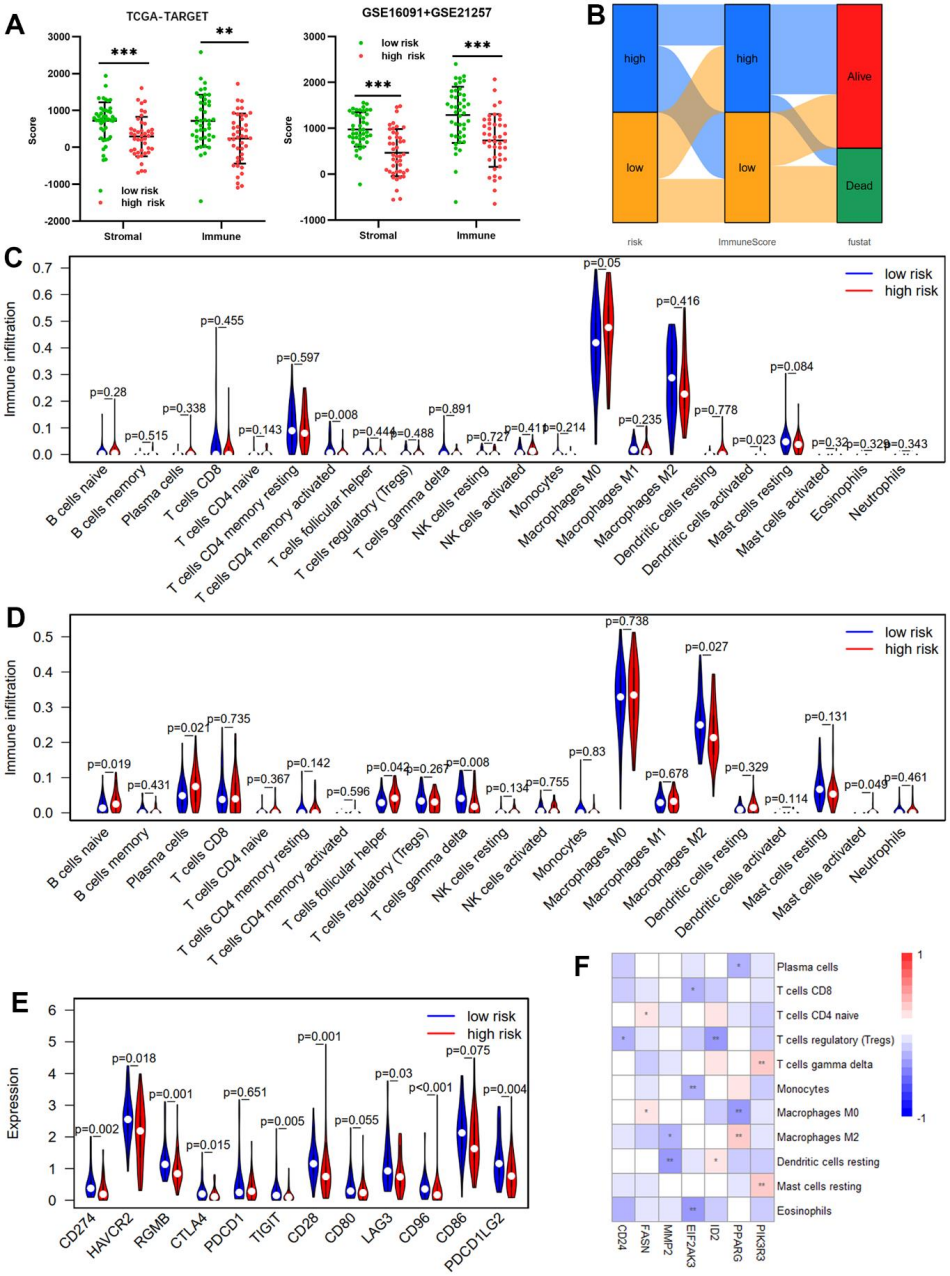
**Immune infiltration between risk groups.** (**A**) Comparisons between the high- and low-risk groups in terms of stromal score and immune score in the TCGA-TARGET cohort and GSE16091 + GSE21257 cohort. (**B**) The Sankey diagram of risk score, immune score, and survival status. Violin plot comparing the proportions of TIICs associated with low- and high-risk scores designated by the OS-prediction model for the training cohort (TCGA-TARGET) (**C**) and the validation cohort (GSE16091 and GSE21257) (**D**). (**E**) Differences in expression of immune checkpoint genes between low- and high-risk groups in TCGA-TARGET cohort. (**F**) Correlation heatmap of prognostic ARGs and TIICs.

Because the tumor microenvironment (TME) influences malignant tumor progression [26], we used the ESTIMATE and CIBERSORT tools to assess the differences between risk groups in immune cells and immune infiltration. Both the validation (GSE16091 and GSE21257) and training (TCGA-TARGET) cohorts showed that the high-risk group had lower immune and stromal scores than the low-risk group, according to the ESTIMATE results (Figure 6A). Low immune scores correspond with low survival rates (Figure 6B). We then compared TIIC differences between risk groups. Results from the TCGA-TARGET cohort revealed that the high-risk group had considerably fewer infiltrating T cells CD4 memory activated than the low-risk group (Figure 6C). GSE16091 and GSE21257 cohort results showed that the high-risk group had significantly fewer infiltrating T cells gamma delta and M2 than the low-risk group, whereas the high-risk group had substantially higher T cells follicular helper, mast cells activated, plasma cells, and infiltrating B cells naïve (Figure 6D). Differences in immune checkpoint gene expression between risk groups were also investigated. In the TCGA-TARGET cohort (Figure 6E), the high-risk group had significantly lower mRNA levels of RGMB, CTLA4, CD96, CD274, HAVCR2, LAG3, TIGIT, CD28, and PDCD1LG2 than the low-risk group. Moreover, the seven OS-related ARGs were associated with different TIICs infiltrating (Figure 6F).

### OGT knockdown inhibited the invasion and migration capability of osteosarcoma cells

**Figure 7:**
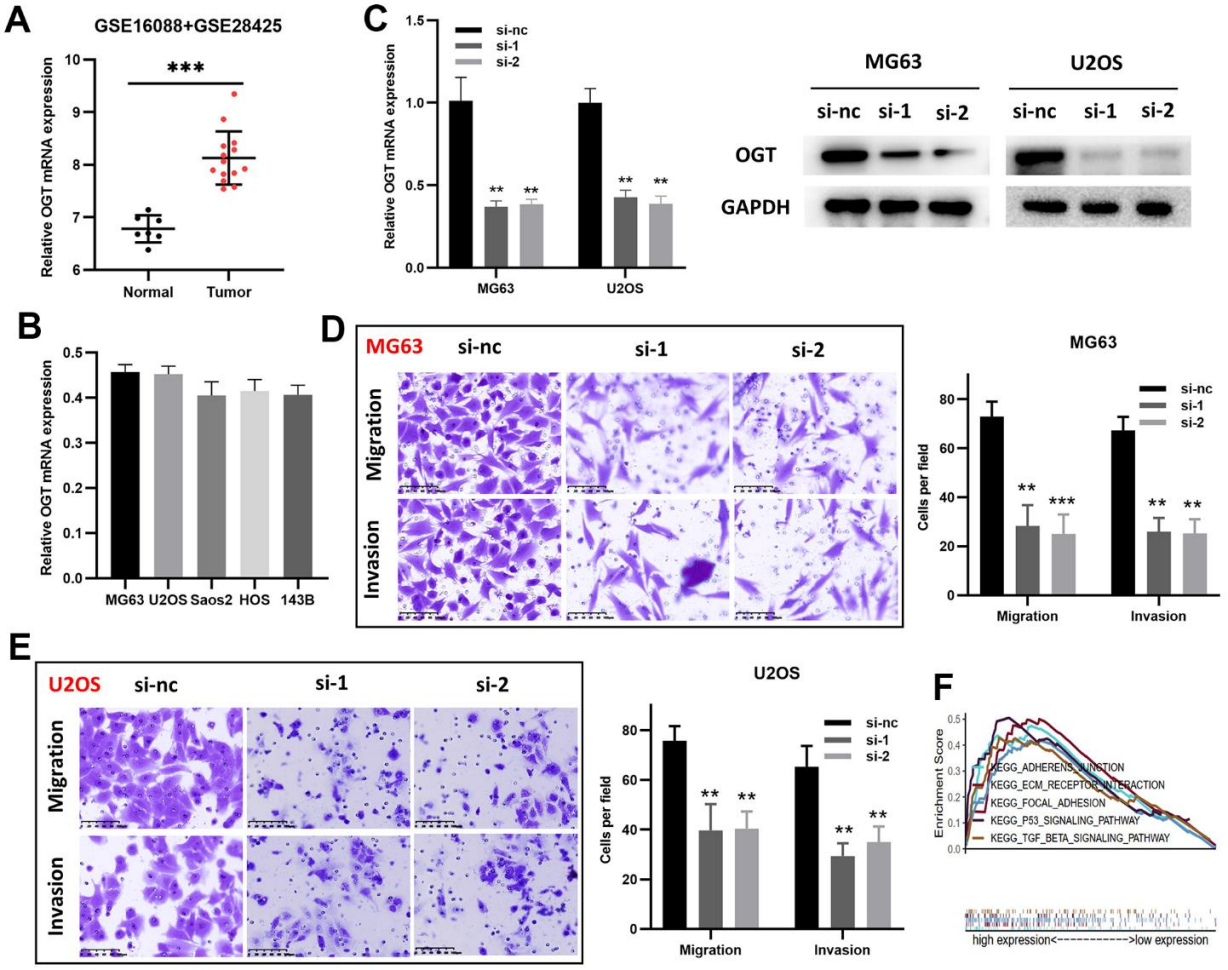
**OGT knockdown inhibits the migration and invasion capability of osteosarcoma cells.** (**A**) OGT expression in normal bone tissues and osteosarcoma tissues (GSE16088 and GSE28425). (**B**) OGT mRNA expression level in osteosarcoma cells. (**C**) OGT knockdown in MG63 and U2OS cells was confirmed using RT-PCR and WB. (**D**, **E**) Migration and invasion capability of MG63 and U2OS cells were significantly weakened by the downregulation of OGT expression (**p <0.01, ***p <0.001). (**F**) KEGG pathway analysis of OGT in osteosarcoma.

Although O-linked N-acetylglucosamine transferase (OGT) has been identified as an ARG associated with patient prognosis, its functions in osteosarcoma remain unclear. Therefore, OGT’s effects on osteosarcoma cells were investigated *in vitro*. Compared to normal bone tissues, tumor tissues had higher OGT mRNA expression levels (Figure 7A and Supplementary Table 2). RT-PCR was used to measure OGT mRNA expression in various osteosarcoma cell lines (Figure 7B). The mRNA level of OGT was high in multiple osteosarcoma cell lines. siRNAs (si-1, si-2, and si-nc) were then used to transfect MG63 and U2OS cells. (Figure 7C). Subsequently, transwell assays revealed that OGT knockdown decreased MG63 (Figure 7D) and U2OS (Figure 7E) cell migration and invasion. OGT-related pro-tumor mechanisms were also investigated using KEGG pathway analysis (Figure 7F). In summary, OGT promoted osteosarcoma metastasis and might be a prognostic biomarker.

## DISCUSSION

Anoikis plays an essential role in cancer progression and has piqued the interest of cancer researchers. Studies on anoikis’ effects on cancer are becoming more common [27, 28], and these studies have allowed us to better understand anoikis’ potential in cancer treatment. ARGs and anoikis have not been thoroughly studied about osteosarcoma. Therefore, a thorough investigation of the functions that anoikis and ARGs exert will aid in the development of novel, individualized osteosarcoma therapy approaches and improve patient prognoses.

Initially, we identified 71 differentially expressed ARGs from 434 ARGs. Cytoscape also identified seven hub genes: *FN1*, *CD44*, *HRAS*, *TP53*, *PPARG*, *CTNNB1*, and *VEGFA*. *FN1* [29], *CD44* [30], *HRAS* [31], *TP53* [32], *CTNNB1* [33], *PPARG* [34], and *VEGFA* [35] genes have been shown to affect osteosarcoma progression.

We then examined the prognostic significance of these differentially expressed ARGs. Nine ARGs were linked to OS, and eight were linked to RFS, according to KM analysis. LASSO regression analysis identified *CD24*, *FASN*, *MMP2*, *EIF2AK3*, *ID2*, *PPARG*, and *PIK3R3* genes as OS prediction model features, and *BRMS*, *COL4A2*, *FGF2*, and *OGT* genes as RFS prediction model features. Based on the ROC analysis, the validation cohort confirmed the OS and RFS prediction models’ ability in identifying osteosarcoma patients. Moreover, univariate and multivariate Cox analyses revealed that RS was an independent OS and RFS risk factor for patients in the TCGA-TARGET dataset. All these findings indicated the clinical value of both models. Finally, we developed OS and RFS prediction nomograms that aid in the more accurate prediction of patient survival.

We then examined hub and prognosis-related ARGs’ potential roles in osteosarcoma and their mechanisms. GO enrichment analysis revealed that the seven hub ARGs were primarily enriched in fibroblast proliferation, cell growth, the transcription regulator complex, protein-C terminus binding, and phosphatase binding, while the prognosis-related ARGs were mainly enriched in lipid metabolic process and smooth muscle cell proliferation. KEGG pathway analysis indicated that the seven hub ARGs primarily participated in virus infection and some cancers, while the prognosis-related ARGs were enriched in the AMPK, AGE-RAGE, and Relaxin signaling pathways. In addition, GSVA analyses suggested that the Ribosome pathway and lysine degradation pathway were the main reasons for the different prognosis of patients with high- and low-risk.

Given immune microenvironment’s involvement in tumors, we further investigated the ARGs-based risk score’s effect on tumor immunity. By comparing the high-risk group’s stromal and immune scores to those of the low-risk group, ESTIMATE showed that the high-risk group had considerably attenuated immune responses. Results of CIBERSORT suggested that risk score affected the infiltration proportions of M2, T cells CD4 memory activated, and plasma cells, which were strongly linked to the prognosis of patients with malignant tumors [20, 36, 37]. Macrophages are the primary immune cell in the microenvironment of osteosarcoma [38]. Thus, regulating macrophages in the TME is critical for improving osteosarcoma prognosis. We also found that PPARG expression was negatively associated with M0 and positively associated with M2. Moreover, PPARG promoted macrophage polarization to the M2 phenotype while inhibiting polarization to the M1 phenotype [39, 40]. As a hub ARG and a prognosis-related ARG, the effect of PPARG on macrophages in osteosarcoma remains unclear and is worth exploring further. Furthermore, CD24 is a novel target for cancer-related immunotherapy [41]. According to this study, CD24 is upregulated in tumor tissues and is associated with OS. Previous research has shown that CD24 is primarily expressed in tumor cells and promotes osteosarcoma invasion and metastasis [42, 43]. Therefore, targeting CD24 in osteosarcoma is a promising therapeutic strategy.

Among these prognosis-related ARGs, the roles and mechanisms of several genes in osteosarcoma have been identified. But the role of OGT in osteosarcoma remains unknown. We showed that OGT knockdown inhibited osteosarcoma cell invasion and migration *in vitro*. KEGG analysis suggested that OGT was enriched in focal adhesion, p53, and TGF-β signaling pathways strongly, which are linked to tumor metastasis [44, 45]. The mechanism of the oncogenic effect of OGT in osteosarcoma remains to be further verified. Nevertheless, OGT has the potential to serve as an osteosarcoma prognostic biomarker.

Our study, however, had some limitations. First, this study focused primarily on mRNA levels, however, protein levels may significantly impact cancer occurrence and progression. Second, the mechanism of action of OGT has not yet been elucidated.

In conclusion, we thoroughly investigated the prognostic significance of ARGs and their link to tumor immunity in osteosarcoma. The OS and RFS prediction models were developed and validated to predict the prognosis of patients with osteosarcoma accurately. We identified hub and prognosis-related ARGs and explored their underlying mechanisms. OGT may serve as a novel target to develop new therapeutic approaches and enhance the prognoses of osteosarcoma patients.

## Supplementary Material

Supplementary Figures

Supplementary Tables
