# Supplementary material for: The anoikis-related gene signature predicts survival and correlates with immune infiltration in osteosarcoma
Source: Aging (Albany NY). 2024 Jan 12;16(1):665–84. doi: 10.18632/aging.205411 (PMC10817411; doi:10.18632/aging.205411)
Supplement: Supplementary Figures [file aging-16-205411-s001.pdf]

SUPPLEMENTARY FIGURES

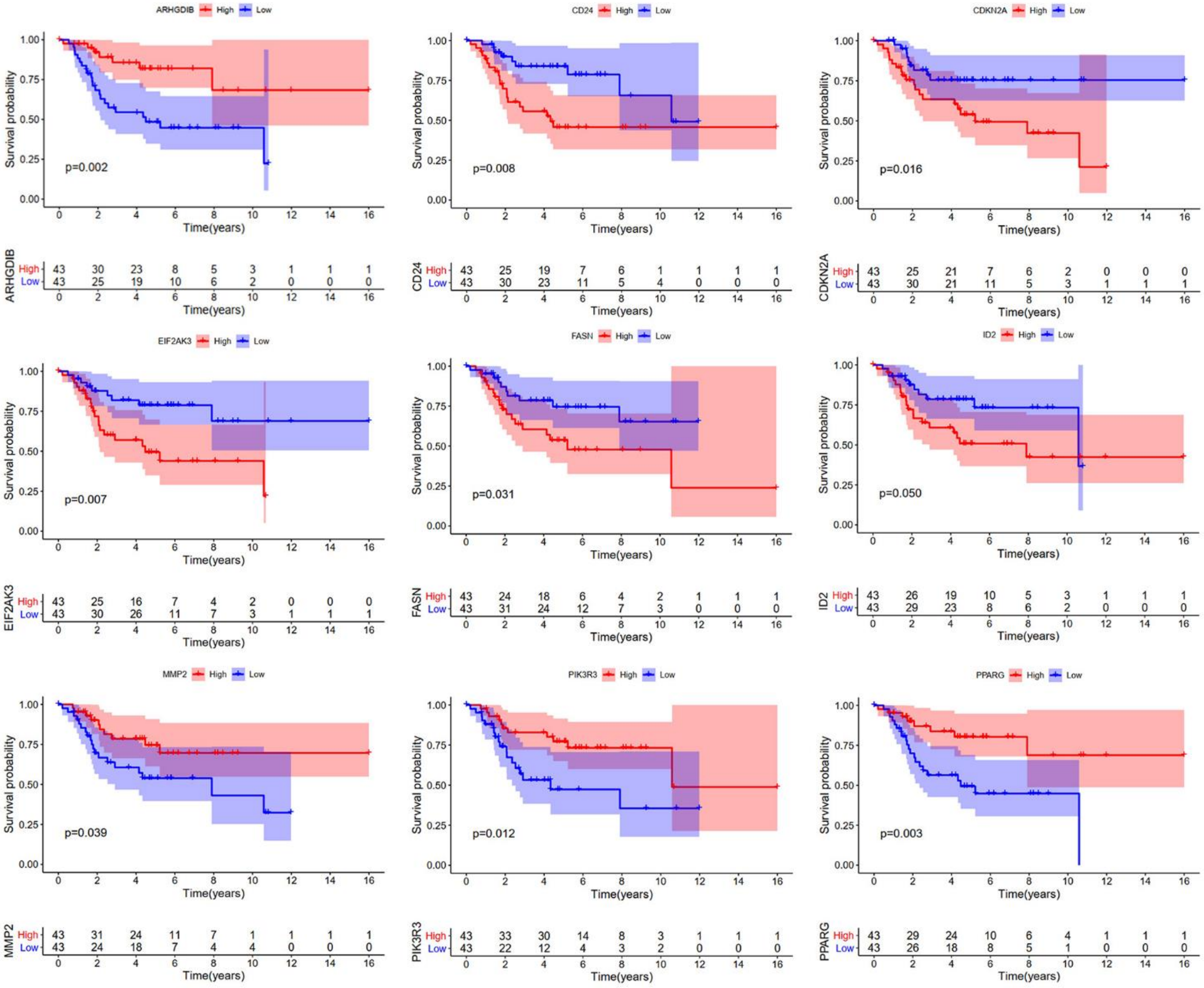

Supplementary Figure 1. Kaplan-Meier analysis of differentially expressed ARGs for OS in TCGA-TARGET.

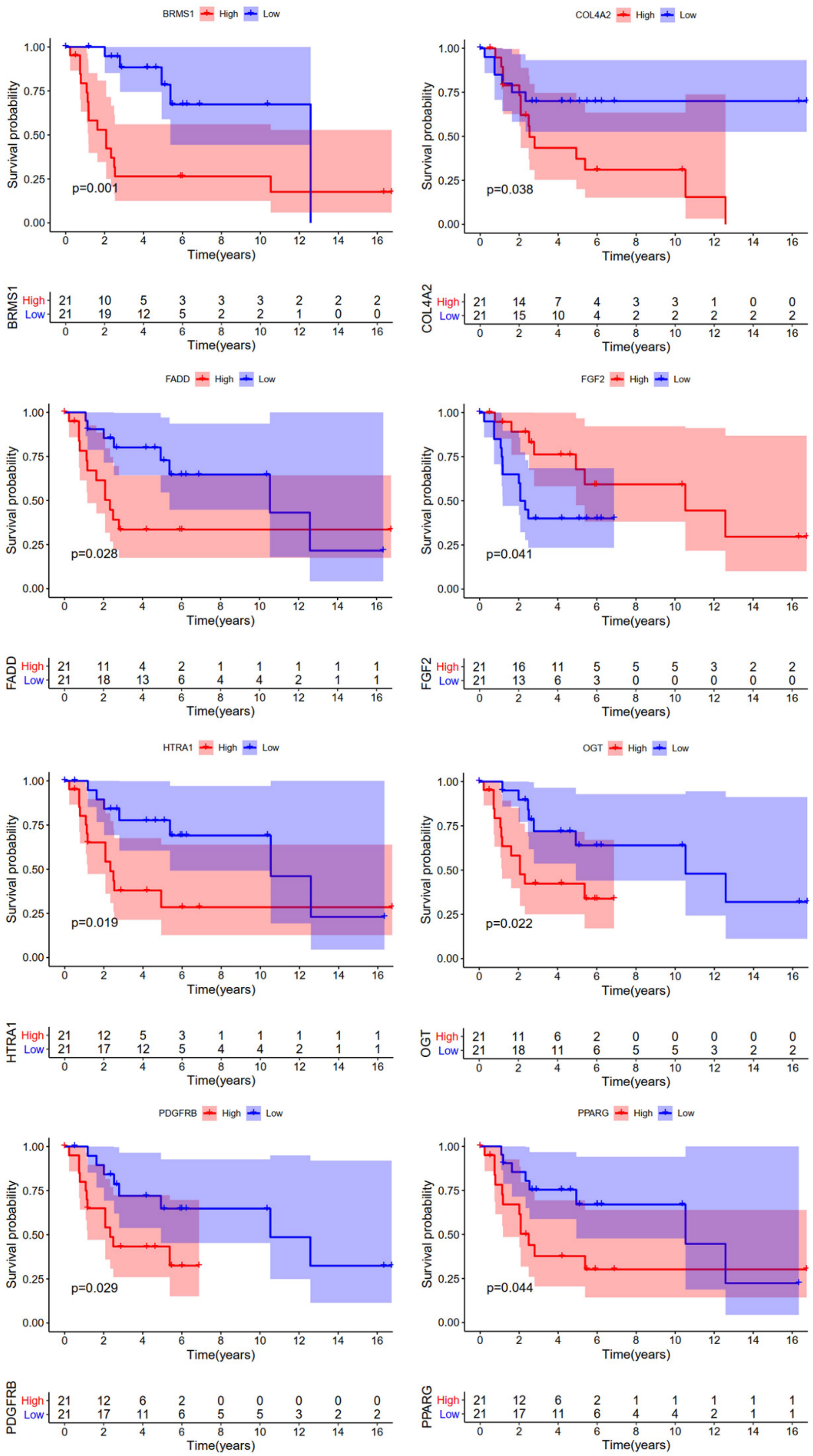

Supplementary Figure 2. Kaplan-Meier analysis of differentially expressed ARGs for RFS in GSE39058.

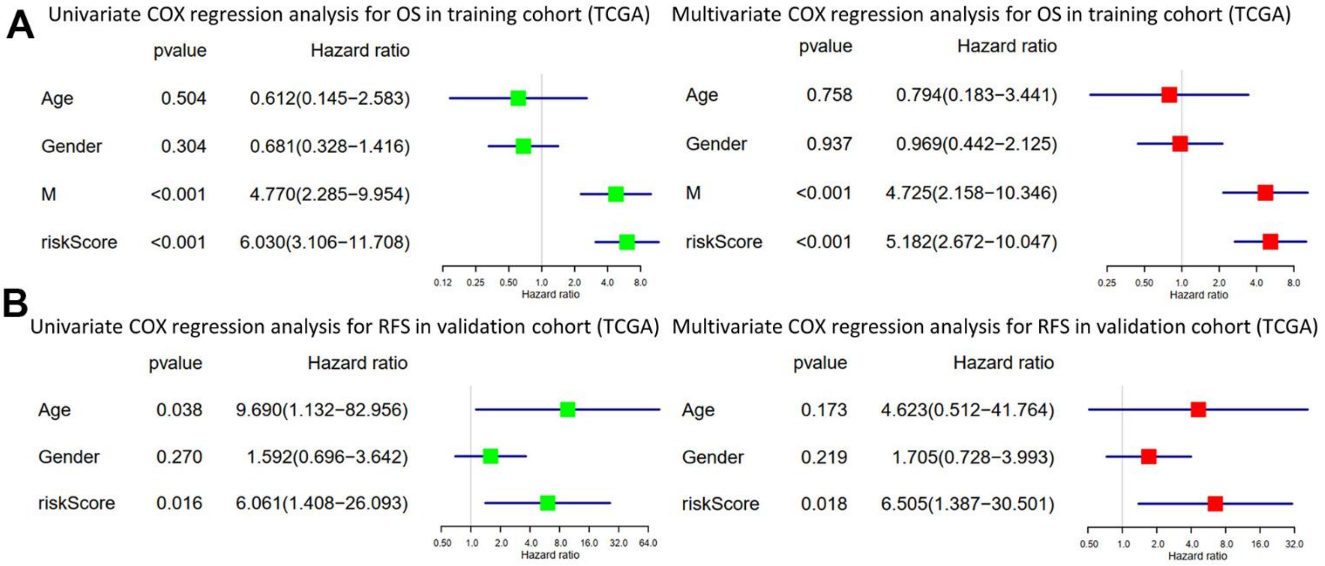

Supplementary Figure 3. Univariate and multivariate Cox analyses for OS (**A**) and RFS (**B**) in TCGA-TARGET.
