# Supplementary material for: The anoikis-related gene signature predicts survival and correlates with immune infiltration in osteosarcoma
Source: Aging (Albany NY). 2024 Jan 12;16(1):665–84. doi: 10.18632/aging.205411 (PMC10817411; doi:10.18632/aging.205411)
Supplement: Supplementary Tables [file aging-16-205411-s002.pdf]

SUPPLEMENTARY TABLES

Supplementary Table 1. The sequences of primer and siRNA oligonucleotides.

OGT	F	5’- TCCTGATTTGTACTGTGTTCGC -3’
	R	5‘- AAGCTACTGCAAAGTTCGGTT -3’
GAPDH	F	5′-CGACCACTTTGTCAAGCTCA-3’
	R	5’-GGTTGAGCACAG GGTACTTTATT-3’
si-1		5′- UAUUUGGAGGUAGUAGUACAA -3′
si-2		5′- UGCAUAAGGUGAGAAGUAGGA -3’

Supplementary Table 2. Differentially expressed ARGs.

gene	logFC	AveExpr	P.Value
ITGA3	-2.465898786	6.113783476	0.00000000000000011
SPP1	6.902439143	11.05799052	0.00000000000000107
TLN1	-2.251123286	7.263180143	0.00000000000000112
OCLN	-1.245569071	5.026531762	0.00000000000000598
HMGA1	-2.817400857	6.881310619	0.00000000000147
FGF2	-1.430950357	5.027533381	0.00000000000414
EIF2AK3	2.518235429	7.62467319	0.0000000000137
QSOX1	-1.556304071	7.836194524	0.0000000000161
HRAS	-2.189618571	7.507511857	0.0000000000597
NQO1	-2.764201857	7.500085619	0.000000000131
PLK1	-1.814372357	7.072995571	0.000000000153
CXCR4	2.932619286	8.121584952	0.000000000235
ARHGDIA	-1.003356357	7.691321905	0.000000000466
CTNNB1	2.029151357	10.013856	0.0000000014
FN1	1.438834429	9.618805952	0.00000000488
SNAI2	3.218966786	9.351329143	0.00000000829
TUBB3	-1.639763143	9.693211143	0.0000000136
MMP9	5.012096214	11.62029619	0.0000000159
FADD	-1.760714429	7.823750095	0.0000000176
SATB1	2.278084071	7.506011	0.0000000233
BRMS1	-1.107130286	7.677278286	0.0000000524
BDNF	-1.434814929	5.661685286	0.0000000616
PIK3R3	1.257319429	6.691640667	0.0000000696
MMP2	2.742156357	9.746927524	0.000000246
PIK3R1	1.443707357	6.869808	0.000000318
CDKN2A	1.8287975	6.899833619	0.000000391
ZEB2	2.18047	6.788587381	0.00000065
FASN	-1.039120571	7.49877681	0.000000704
CAV1	-2.043785714	8.664928048	0.000000752
OGT	1.3462075	7.679012952	0.000000951
ARHGDIB	2.196897786	9.578011667	0.00000139
PIK3C2B	1.339768286	7.115673429	0.00000153
HTRA1	2.940662929	9.721752381	0.00000162
S100A4	2.498585714	11.29989838	0.0000019

PPARG	-1.439115929	6.189688952	0.00000201
SERPINE1	-2.181896929	6.826633619	0.0000023
TNC	2.953049714	9.317173381	0.00000232
ID2	1.409741929	8.227120381	0.00000276
CDKN3	-1.849537714	8.312781714	0.00000278
LGALS3	1.438387143	9.974514619	0.00000287
VEGFA	1.855724429	8.161938333	0.00000307
CD24	2.703499071	8.175575571	0.00000372
TP53	-1.408055643	5.80863981	0.00000435
PDGFRB	1.772347286	8.673447	0.00000527
TFDP1	-2.144229	8.82953	0.00000624
PRDX4	1.154507357	11.17791057	0.00000779
MMP13	4.048848071	8.890155333	0.0000134
TWIST1	2.904488	8.616746762	0.0000177
TNFSF10	1.447986857	6.320476571	0.0000205
IFI27	2.892314643	9.11628019	0.0000286
ITGAV	1.488104643	9.616141048	0.0000407
GSK3B	-1.110349357	7.996281238	0.0000408
RHOB	1.162412929	9.141598524	0.0000418
KDR	1.211653071	6.197537857	0.0000562
MTDH	1.142134143	8.96726719	0.0000893
BSG	1.288856286	9.129787667	0.000111583
INHBB	1.4292265	7.336986238	0.000113616
LAMB3	-1.244247143	5.92900219	0.000118277
CDKN1B	1.025396429	8.705005905	0.000124414
CD36	1.936409786	7.407610857	0.000245353
UBE2C	-1.017923429	9.383675524	0.000247511
YAP1	-1.002773214	6.590675381	0.000271862
CD44	-1.131310571	7.854753095	0.000274192
SFN	-1.500931857	7.158165476	0.000302139
PRPF4B	1.0970545	7.460391238	0.000344657
PBK	-1.576046643	7.686079381	0.00051086
ANGPTL2	1.468309143	8.121412333	0.000683007
COL4A2	1.056079786	9.235457619	0.002313379
CSPG4	1.066916643	7.449595619	0.002932988
TPM1	-1.061697786	9.346862476	0.003099409
HMOX1	1.137150643	8.249676857	0.005940539
